# Expanding family medicine in Latin America: health system drivers, workforce trends, and policy priorities for strengthening Primary Health Care

**DOI:** 10.3389/fmed.2026.1870793

**Published:** 2026-08-17

**Authors:** Shirley Esneda Mendoza-Rincón, Ma Gpe Alvarado-Rodríguez, Fátima Liz González-Ayala, Lusy Paulyna Orellana-Navarrete, Rita Inés Trujillo-Sagel, Kelly De la Cruz-Torralva, Eduardo Luis Caycedo-Guevara, Maria Sofia Cuba-Fuentes, Mauricio Alberto Rodríguez–Escobar

**Affiliations:** 1 Colombian Society of Family Medicine, Bogota, Colombia; 2 Universidad El Bosque, Bogota, Colombia; 3 Facultad de Medicina, Universidad Autónoma de San Luis Potosí, San Luis de Potosi, Mexico; 4 Facultad de Ciencias Médicas, Universidad Nacional de Asunción, Asunción, Paraguay; 5 Paraguayan Society of Family Medicine, Asunción, Paraguay; 6 Pontificia Universidad Católica del Ecuador, Quito, Ecuador; 7 Universidad de las Américas, Quito, Ecuador; 8 Panamanian Association of Family Medicine, Panama, Panama; 9 Universidad de Panamá, Panama, Panama; 10 Center for Research in Primary Health Care, Universidad Peruana Cayetano Heredia, Lima, Peru

**Keywords:** family medicine, health workforce, Latin America, policy and practice, primary healthcare

## Abstract

**Background:**

Family Medicine is fundamental to effective Primary Health Care (PHC), yet its development across Latin America has been uneven, constraining access, resolution and equity.

**Objective:**

To synthesize workforce trends and policy drivers shaping the expansion of Family Medicine in seven Latin American countries (2014–2024) and to propose actionable policy and implementation priorities for PHC strengthening.

**Methods:**

Directed multinational mixed-methods review (Argentina, Colombia, Ecuador, Mexico, Paraguay, Panama, Peru). We integrated a structured search of peer-reviewed and gray literature, official documents and national workforce registries with secondary data analysis (standardized rates per 100,000 inhabitants) and country-level validation by national experts from the Ibero-American Network of Family Medicine (IBIMEFA).

**Results:**

All countries showed increases in Family Medicine specialists, with heterogeneous magnitude and pace, rapid expansion in Ecuador and Paraguay; steady growth in Colombia and Peru; incremental increases in Mexico and Argentina; and nascent development in Panama and slower workforce growth in Panama despite a well-established specialty training system. Enablers included expansion of residency capacity, explicit PHC policy prioritization, and university service partnerships. Key barriers were fragmented financing and organization, inequitable geographic distribution of specialists, and weak formalization of Family Medicine as the system’s entry point.

**Conclusion:**

The increase in family medicine specialists in the region calls for integrated policies on training, funding, and organization to translate these advances into sustainable improvements in quality, problem-solving capacity, and equity; evaluative studies are needed to guide implementation. Employability, and service organization to translate these advances into sustainable improvements in quality, problem-solving capacity, and equity. Beyond workforce expansion, strengthening the professional identity of family physicians and their integration within communities may contribute to the long-term sustainability of health systems and Primary Health Care. Further evaluative studies are needed to guide policy implementation and assess impact.

## Introduction

Family medicine specialists play a key role in implementing the Primary Health Care (PHC) strategy within the health care system. They are regarded as the central professionals at this level of care and share common elements with the principles and functions of primary health care, such as accessibility, comprehensiveness, continuity, and a family- and community-centered approach, among others (1).

Family Medicine emerged during the second half of the twentieth century as a response to increasing medical specialization, fragmentation of care, and the need to strengthen person-centered healthcare. Unlike organ- or disease-focused specialties, Family Medicine developed as a discipline centered on the individual, the family, and the community, integrating biological, psychological, and social dimensions of health across all stages of life. Its consolidation was closely linked to the development of Primary Health Care and was reinforced by international initiatives promoting equitable and comprehensive health services, particularly following the Alma-Ata Declaration and subsequent global efforts to strengthen health systems through PHC (2–4).

Family medicine focuses on providing comprehensive and continuous care to patients throughout their lives, addressing everything from prevention to the management of a wide range of health issues in a context-sensitive and highly effective manner. Evidence suggests that robust primary care systems, with a significant presence of family physicians, can contribute to better population health, greater patient satisfaction, reduced use of specialty services, and lower healthcare costs (2–4).

In Latin America, the development of Family Medicine has been gradual and closely linked to broader health system reforms aimed at strengthening PHC. However, this process has been heterogeneous across countries, shaped by differences in governance, financing structures, training capacity, and institutional support. Despite recent growth in the number of specialists, persistent challenges remain, including workforce shortages, uneven geographic distribution, fragmented health systems, and limited integration of family physicians into service delivery models (5, 6). Although the importance of Family Medicine is widely recognized, there is limited comparative evidence analyzing trends in specialist growth alongside structural health system characteristics and policy drivers across countries in the region. Most available studies are country-specific or narrative, lacking a standardized regional perspective.

This study addresses this gap by analyzing trends in the number of Family Medicine specialists and identifying key factors associated with their growth in seven Latin American countries between 2014 and 2024. By integrating documentary evidence with expert validation, this study aims to provide a comparative regional perspective to inform policies for strengthening PHC systems.

## Scope and methods

We conducted a directed multinational mixed-methods review to synthesize trends in Family Medicine workforce development in seven Latin American countries (Argentina, Colombia, Ecuador, Mexico, Paraguay, Panama and Peru) between 2014 and 2024. Data sources included a structured literature search (PubMed, Scopus, Web of Science, LILACS, Google Scholar), official documents, national workforce registries and gray literature. We extracted indicators on numbers of practicing specialists, trainees, residency duration/structure, roles of family physicians and select health system features; indicators were standardized per 100,000 inhabitants. Country-level findings were triangulated with input and validation from national experts affiliated with the Ibero-American Network of Family Medicine (IBIMEFA), who reviewed country data and provided contextual interpretation. Given heterogeneity in definitions and sources, the study uses descriptive comparative analysis to prioritize comparability; it does not support causal inference.

### Design

A multinational descriptive comparative study was conducted based on a structured review of scientific literature, official documents, professional registries, and other documentary sources, complemented by validation from national experts. The study aimed to characterize and compare the development of Family Medicine in seven Ibero-American countries and to identify factors associated with the growth of the specialty between 2014 and 2024.

Countries and study variables:

The study included Argentina, Colombia, Ecuador, Mexico, Panama, Paraguay, and Peru.

Information was collected on the following domains:

- Health system characteristics and organization of Primary Health Care (PHC).
- Institutional development of Family Medicine within each country.
- Number of practicing family physicians.
- Number of physicians in specialty training programs.
- Duration and characteristics of residency training.
- Academic and institutional support for the specialty.
- Professional roles and scope of practice of family physicians when such information was available.
- Trends in workforce growth and training opportunities.

### Data sources and search strategy

A structured search was performed in indexed and non-indexed sources, including PubMed, Scopus, Web of Science, LILACS, and Google Scholar. Additionally, official documents from ministries of health, universities, residency programs, scientific societies, professional associations, workforce registries, and governmental reports were reviewed.

The main search period covered publications and reports from 2014 to 2024. When contemporary information was unavailable or incomplete, earlier sources were reviewed to provide historical context and document long-term trends in the development of the specialty.

Search terms were used in Spanish, Portuguese, and English and included:

“Family Medicine” “Family Physician” “Family Practice” “Medicina Familiar” “Medicina de Familia” “Medicina General” “Medicina de Família e Comunidade” “Primary Health Care” “Workforce” “Residency Training” “Medical Specialization.”

These terms were combined with the name of each participating country using Boolean operators when supported by the database.

### Eligibility criteria

Priority was given to peer-reviewed scientific publications and official documentary sources.

Sources were included when they provided information related to at least one of the study variables, including workforce data, training capacity, characteristics of health systems, organization of PHC, or institutional development of Family Medicine.

Gray literature, professional registries, reports from scientific societies, university documents, and journalistic sources citing official data were considered only when official or peer-reviewed information was unavailable or incomplete. In all such cases, information was subjected to additional verification through documentary triangulation and expert review.

Sources lacking sufficient methodological transparency, clear institutional origin, or consistency with other available evidence were excluded.

### Data extraction and validation

Data were extracted using a standardized framework developed by the research team. Information was organized according to predefined analytical categories:

1. Health system classification.
2. Organization of Primary Health Care services.
3. Institutional origin and development of Family Medicine.
4. Training programs and residency opportunities.
5. Family physician workforce and recent growth trends.
6. Professional roles and functions of family physicians.

Information obtained from different sources was compared and cross-checked to identify inconsistencies. When discrepancies were identified, priority was given to official records and consensus-based validation.

Country-specific information was reviewed by national experts from the IBIMEFA/CIMF network who were members of their respective Family Medicine scientific societies. Experts verified the consistency of the data, provided contextual interpretation, and helped resolve discrepancies identified during the document review process. In Panama, where historical information was scarce, additional input from key informants was used to complement documentary sources.

### Data analysis

Data were synthesized by country and analyzed through descriptive comparative methods. Indicators related to workforce size, specialist training, institutional development, and health system organization were compared across countries.

Temporal trends between 2014 and 2024 were described whenever sufficient information was available. For some countries, historical data from periods preceding 2014 were incorporated to contextualize workforce evolution or address gaps in official records. Any discrepancies among sources were documented and resolved through triangulation and expert consensus.

## Results

Data were collected from seven countries: Argentina, Colombia, Ecuador, Mexico, Paraguay, Panama, and Peru.

To assess the development of Family Medicine in the region, four analytical areas are presented: (1) Health system structures and financing; (2) Primary care organization and role of family physicians; (3) Origins, training capacity and institutional support for Family Medicine; and (4) Growth in Family Medicine workforce 2014–2024.

## Health system structures and financing

This review considered types of coverage (public/private) and the presence of insurance mechanisms, using the 2019 classification (5) and recent literature, with expenditure figures standardized according to the World Bank (2021) (7) to ensure comparability. The countries analyzed exhibit diverse institutional structures, shaped by their political history, economy, and social protection models, which result in different modes of financing, organization, and delivery (Table 1).

**Table 1:** Health system structures and financing.

Country	Type of system	Relationship between the government and private providers	Administrative decentralization	Total, health expenditures (% GDP, 2021)
Argentina	Mixed system: public, social security, and private. High fragmentation among levels and subsystems (8)	There are prepaid plans and private health insurance providers (mutuals) for those who voluntarily enroll in these plans (Health insurance plans) (8).	Significant provincial autonomy (8).	10.04% (7).
Colombia	Universal insurance system (9).	Private insurers manage public funds. With private contributions providing supplementary coverage, out-of-pocket expenses are not reported to be very high (9).	Decentralization with variable capacities (9).	9.02% (7).
Ecuador	Mixed system: MSP (provides care to people without health insurance), IESS (covers workers and their families), and private (11).	Mixed procurement of service providers. High participation of private providers with out-of-pocket expenses (11).	Decentralized and partially decentralized (11).	8.21% (7).
Mexico	Fragmented insurance system: insurance schemes, government employees, private-sector workers, public health insurance, and others (12, 16).	Primarily public provision, with some private providers (12, 16).	High institutional fragmentation (12, 16).	5.89% (7).
Paraguay	A mixed system with a predominantly public component. A social security subsystem for workers and their beneficiaries (19).	Limited insurance coverage. High out-of-pocket costs for private supplemental insurance (19).	Emerging decentralization (19).	8.03% (7).
Panama	A mixed system comprising the CSS (covering workers and their dependents), MINSA (regulatory body and coverage for those not enrolled in the CSS), and the private sector (18)	Coverage is predominantly provided by the public sector	Mixed and Parallel Model with Fragmentation	5.38% (13)
Peru	Segmented system: SIS (covers the non-working poor), EsSalud (covers workers, spouses, and children), and private insurance (14).	Mixed procurement (14).	Decentralization with coordination issues (14).	6.68% (7).

Original work.

The seven countries have mixed or segmented systems in which public services, social security subsystems, and a private sector coexist, with varying degrees of prominence (5, 8–14). Argentina and Mexico exhibit high fragmentation among subsystems and jurisdictions, leading to overlaps, barriers to access, and local variations in the quality and availability of services (8, 12, 15, 16). Peru, Ecuador, and Paraguay also show segmentation between contributory schemes and public programs, with negative effects on continuity of care and equity (5, 11, 14, 17). Colombia, although structured as a state-regulated universal insurance model, is notable for its management of resources through private insurers, while Panama operates with two parallel public networks plus the private sector, which complicates functional integration (9, 13, 18).

In all countries, the private sector acts as a complement to the public system, but its relative weight and households’ reliance on out-of-pocket spending vary; in some countries, the private sector meets the demand of those who can afford more specialized services, while in others, mixed procurement and fragmentation increase the burden of out-of-pocket spending and hinder financial protection (9–12, 19, 20).

Decentralization is a recurring theme, but its implementation produces varying effects; high subnational autonomy in countries such as Argentina and Mexico amplifies territorial disparities, and in countries such as Peru and Ecuador, coordination limitations between the central government, social security, and local governments hinder reforms aimed at strengthening primary care and continuity of care (8, 14–17, 21, 22).

This reveals patterns in these countries characterized by persistent institutional fragmentation that limits integration and continuity of care; subnational heterogeneity stemming from varying degrees of decentralization; and the absence of a direct relationship between higher levels of spending and better outcomes in terms of equity or financial protection, given the significant role of private financing and out-of-pocket spending in various contexts (8, 9, 11–18, 20–24).

As can be seen, the health systems of these seven countries feature diverse institutional structures shaped by their political history, economic structure, and social protection models, which result in distinct arrangements for financing, organization, and service delivery. In practice, this manifests as heterogeneous coverage across population groups (for example: the population served by state services versus workers and their families covered by social security systems), which leads to segmented delivery structures (different populations covered by distinct schemes: public, social security, private) and frequently fragmented structures (multiple actors, duplication of benefits, gaps in the continuity and coordination of services).

## Primary care organization and role of family physicians

Organizing service delivery poses a challenge for the implementation of PHC. Regarding service delivery within networks, there is discussion of the importance of gateway services, referred to in European and North American literature as Primary Care. The collection of this information was also based on a classification used in a 2019 publication (25), supplemented with information from family physicians in the participating countries. Difficulties were also encountered in collecting the data, particularly regarding how to define and interpret the terms used and determine whether the various criteria were met.

This section provides a comparative summary, by country, of key characteristics of primary care, including the size of the assigned patient population, team-based organization, a resolution rate of over 80%, and the involvement of family medicine specialists at the point of entry; other relevant aspects not included in the analysis are accessibility, person-centered care, and mechanisms for coordination with the healthcare network (3, 26) (Table 2).

**Table 2:** Primary care and its organization, and the involvement of family physicians.

Country	Territorial affiliation (dependent population)	Formation of Primary Health Care teams	First-level resolution	Family physician as the first point of contact
Argentina	Partially. Known as the dependent population or georeferenced population; it varies by province.	Existing primary health care teams, but with varying coverage and capacity; this varies by province.	Variable resolution, depending on provincial capabilities; there is no uniform figure.	Proposed with little progress; no distinction between general medicine and family medicine; few financial incentives.
Colombia	Documents and policies specify the population assigned to each team; in practice, the assignment varies and is often based on the insurer.	The Comprehensive Care Policy calls for primary care teams; implementation varies across regions and insurers.	Variable effectiveness; depends on regional capacity and resources; mandated by regulations, but with gaps in implementation.	It is not always explicitly identified in the regulations as the sole point of entry; official documents outline its role.
Ecuador	In urban areas, there is approximately one unit per 4,000 residents; in rural areas, there is approximately one unit per 2,500 residents (uneven distribution).	Recent process; PHC teams have been established, but previous models still coexist; implementation has been uneven.	An emerging process; the coexistence of different models limits the ability to uniformly measure resolutivity.	A family physician has been nominated as a primary care specialist; there is a significant shortage of specialists.
Mexico	In many cases, patients are assigned based on their insurance plan or insurer rather than by geographic area; there are local assignment models.	Primary healthcare teams at IMSS, ISSSTE, and MINSA, and programs such as IMSS-Bienestar; variations among states.	The extent of coverage depends on the user’s insurance plan; initial assessment of limitations.	The role of the family physician varies; in some subsystems, they serve as the primary point of contact, while in others their role is less prominent (estimated partial coverage: 50%).
Paraguay	There is an intention to enroll participants and expand coverage gradually, but actual coverage is limited and enrollment is partial.	Primary Health Care (PHC) programs are in place, but with insufficient resources and coverage. Within the PHC Program: Standardized Family Health Teams (FHTs), with sustained expansion funded by public resources, although infrastructure gaps remain in rural areas.	Limited resolution capacity and access; shortages of human resources and access in rural areas.	The goal is to incorporate family physicians into primary care; current coverage is low.
Panama	Mixed enrollment models between CSS and MINSA; mobility between systems; territorial enrollment defined in local programs.	Primary Health Care Programs at the Ministry of Health and the Social Security Institute; regional and facility-level variation.	Mixed results; progress in program integration, but with regional disparities.	Recognized in policy circles and academia, family medicine is present in top-tier programs and centers, but with significant variation.
Peru	MINSA/SIS and EsSalud models with defined coverage; variable team size (estimated at 400–800 people per team in some areas).	Primary Health Care (PHC) teams at the Ministry of Health (MINSA) and EsSalud; implementation and varying coverage; proposals for strengthening these teams since 2008.	Accountability is set as a goal but is limited by funding and resources; indicators are incomplete	In academic settings, it is often presented as an entry point; in top-tier institutions, it is typically viewed as a specialized supplement.

Adapted from Rodríguez et al. (6).

The comparative analysis reveals that the countries examined have made policy and programmatic progress in terms of population enrollment and the establishment of primary care teams; however, this progress is uneven and, in most cases, has not yet translated into consolidated systems with effectively assigned populations and standardized indicators that enable monitoring, performance evaluation, and ongoing territorial management. There is a prevailing gap between policy design and operational implementation, evidenced by regional variations, differences among subsystems, and human and financial resource constraints (Table 2).

About first-level resolution, none of the seven countries has explicit, uniform, and verifiable targets that define the expected level of resolution at the entry points within service networks. The available information suggests that problem-solving capacity depends more on local or institutional capabilities than on clearly defined national standards. In this context, the family medicine specialist appears more frequently as a projected or desirable figure within the model than as a fully institutionalized and established actor. The recurrence of expressions such as “proposed,” “intended,” “variable presence,” “partial or low coverage,” “heterogeneity,” and “specialized complement” reflects that, in the region, their role is still in a transitional phase from conceptual recognition toward effective structural integration into health systems (Table 2).

## Origins, training capacity and institutional support for family medicine

The origins of family medicine in the countries analyzed are linked to international movements to strengthen primary care in the 1960s and 1970s and to subsequent national health reforms in each country (2, 6). Its official recognition and establishment as a medical specialty occurred gradually (1970s–2000s), driven by universities, government ministries, scientific societies, and social security agencies, factors that have shaped the training and growth of specialists in recent years (25, 27).

Although all seven countries share a common historical connection with PHC-oriented reforms, the institutional trajectories of Family Medicine have differed considerably. Variations in health system organization, government priorities, academic support, and workforce planning have influenced both the pace of development of the specialty and its integration into service delivery models. In general, the expansion of Family Medicine has depended on the establishment of residency programs, the recognition of the specialty by academic and regulatory bodies, and the incorporation of family physicians into primary care strategies and health system reforms (25, 27).

The timing and magnitude of this development have varied across countries. Argentina and Mexico were among the earliest adopters, establishing residency training programs several decades ago and accumulating larger workforces. Ecuador and Paraguay have experienced significant expansion in recent years, largely associated with initiatives aimed at strengthening PHC and increasing training capacity. Colombia, Peru, and Panama have shown more gradual but sustained development, supported by universities, scientific societies, residency programs, and national health institutions. Despite these advances, important differences remain in the number of training positions available, workforce production, institutional support mechanisms, and the role assigned to Family Medicine within health systems (Table 3).

**Table 3:** Growth in the number of family physicians, 2014–2024.

Country	Official launch of the program	Family medicine specialist	Number of family physicians per 100,000 inhabitants	Family medicine specialist	Number of family physicians per 100,000 inhabitants	Family medicine resident/early career	Family medicine resident/recent graduate
Argentina	Años 60–70	2500 (2003) (72)	6,51	6,191 (2015) (73)	14,24	428(2014) (74)	313 (2019) (29)
Colombia	1984 (formal)	623 (2014) (33)	1,31	1128 (2024) (36)	2,13	180 (2016) (5)	523 (2023)^1^
Ecuador	1987	300 (2013) (76)	1,91	2306 (2025) (41)	12,6	600 (2014) (77)	123 (2024) (82)
Mexico	1971 (UNAM + IMSS)	16,895 (2017) (46)	13,54	23,224 (2023) (42)	17,9	1,200(2011) (5)	2,580 (2023) (48)
Panama	1976	40 (2006) (5)		80 (2018–2024) (53)	1,77	sd	8 (2024)^1^
Paraguay	1981	240 (2014) (5)	3,54	664 (2024) (19)	10,42	151 (2014) (5)	510 (2024) (78)
Peru	1980	400 (2015) (79)	1,28	1,205 (2025) (80)	*3,48*	143 (2013) (79)	191 (2025) (81)

1 Information on specialization program coordinators as of 2023.

The following section outlines the development of the specialty in the countries analyzed, the characteristics of training programs, the number of family physicians, and the roles they play in their respective health care systems.

### Argentina

Training in family medicine began in the late 1960s and early 1970s, with residency programs in provinces such as Neuquén, Misiones, Córdoba, Jujuy, and Buenos Aires; the residency system was established by Ministerial Resolution No. 1778/60 and Law No. 22,127 (1979), which created the National Health Residency System, and the specialty was formally recognized in 2006 (Resolution No. 1923/06) (28). Between 1980 and 2000, professional societies and associations emerged, including the Argentine Association of Family Medicine (AAMF), the Argentine Association of General Medicine (AAMG), the Argentine Federation of Family and General Medicine (FAMFyG), and the Argentine Federation of General Medicine (FAMG), which promoted training and research, and in 2008, the Federal Health Council (COFESA) declared the specialty a priority within the Primary Care strategy, facilitating funding for residency positions (28).

The program emphasizes comprehensive and continuous primary care, interdisciplinary work, and responsibility for an assigned patient population; graduates practice in the public sector, the social security system, and the private sector (28). In 2015, there were 6,191 general/family practitioners, accounting for just under 5% of all specialists in the country, with a rate of 14.24 per 100,000 inhabitants (29). And in 2019, there were 313 residents in the specialty; geographic concentration and jurisdictional diversity are distinctive features of the country (28–32).

### Colombia

The history dates back to the late 1960s and 1970s (25) in collaboration with National University of Colombia and the University of Valle; the first officially recognized program was established in 1984 at the University of Valle, offering a three-year degree program (27). With the enactment of Law 100/1993, Family Medicine was recognized within the General System of Social Security in Health (SGSSS) (27, 33). Since 2000, the Ministry of Health and Social Protection (MSPS) has promoted its expansion, and Law 1438/2011 together with the National Unified Program (2013) fostered its standardization and the growth of training programs (33–35).

Family physicians perform clinical roles (primary care, management of chronic diseases, support to healthcare teams, and increasing participation in hospital and emergency settings), as well as administrative, teaching, and research functions; their distribution is conditioned by the organization of the health system, with a predominance in the private sector and variable presence in the public network depending on regional decisions (25, 36, 37). The specialty is taught in universities through three-year programs; in 2023 there were 523 specialists in training, and by 2025 there were 17 programs (35). In 2024, 1,128 Family Medicine specialists were registered, with a rate of 2.13 per 100,000 inhabitants out of a total of 47,990 specialists (38).

### Ecuador

The earliest background dates back to the founding of Hospital Vozandes (Quito) in 1955 and the design of the curricular framework inspired by the American Academy of Family Physicians in 1986; the first residency program began in 1987 at the Catholic University of Cuenca, based at Hospital Vozandes (39, 40). Until 2005, the public sector lacked Family Medicine services; it was not until 2017 that a unified program with a single curriculum was promoted, encouraging the large-scale expansion of the specialty (39–42).

Family physicians perform administrative, teaching, and clinical roles, working in both the public and private sectors throughout the country (39, 42). Training combines hospital and community settings (three-year residency). By 2014, Ecuador had 22 medical schools, of which 8 offered postgraduate training in Family and Community Medicine (4 private and 4 public); in 2024, 123 residents in training were reported (41).

The figures for specialists vary by source. INEC recorded 1,387 specialists in 2017 (with an estimated deficit relative to demand), and later official sources reported between 1,460 and 2,306 specialists depending on the registries and definitions used (42, 43). In 2017, the rate was 8.3 per 100,000 inhabitants (with a projection of 12.6 per 100,000 for 2025) (43).

### Mexico

Mexico was a pioneer in the region, with the IMSS promoting the “family physician” model since 1954; the specialty received university accreditation in 1971 from the National Autonomous University of Mexico (UNAM) and was further consolidated through postgraduate and continuing education programs beginning in 1993 (44–46). Family Medicine developed extensively in primary care units with physicians assigned to specific populations (2,000–3,000 people), although national coverage remains insufficient and the practice has faced challenges due to cuts in benefits and an emphasis on curative care over prevention (47–49).

Family physicians perform clinical, teaching, administrative, research, and managerial duties; compared to general practitioners, they tend to have greater job stability and managerial responsibilities in formal public-sector positions (44, 48, 50, 51).

Training in this specialty is primarily through residency (3 years); in addition to the National Medical Residency Exam (ENARM), there are also in-service specialization programs under the Academic Competency Exam (ECA), which is structured as an in service training program aligned with the curriculum of the Single Plan for Medical Specializations, for physicians affiliated with institutions (52). There are an estimated 23,224 specialists in Family Medicine, with a rate of 17.9 per 100,000 inhabitants, and 2,580 residents in training (45, 48, 50). The IMSS provides 85% of primary care, followed by ISSSTE (9%) and others (46).

### Panama

Family Medicine in Panama was consolidated in the 1970s with the creation of the Department of Family Medicine at the University of Panama and the start of the residency program within the Social Security Fund (6); in 1979 the first two family physicians graduated (6, 53). However, it was not until 2009 that the National Coordination of Family Medicine for Family Medicine specialists within the Social Security Fund was established (54). By 2025, there is a single national university program accredited by the University of Panama, Social Security Institution and the Ministry of Health; the residency lasts 3 years (with 1–2 optional months of “externship”) and is carried out at training sites of the Ministry of Health and the Social Security Fund nationwide (55).

An interesting aspect is that residencies include a contractual obligation to serve twice the duration of training in the assigned location (6 years in Family Medicine), which influences the decision to accept training positions (56). By 2024 there were 80 practicing family physicians and an estimated population of 4,515,577 for that year (1.77 per 100,000 inhabitants), with clinical, administrative, teaching, and research roles, mostly within the Social Security Fund; and there is no universal population assignment to a primary care physician, despite previous attempts (55).

### Paraguay

The specialty began in 1981 with the first residency program at Hospital Bautista (Asunción) and obtained academic recognition in 1991 with the opening of the program at the Faculty of Medical Sciences (25). The Paraguayan Society of Family Medicine (SPMF, 1990) and the Pan American Health Organization (PAHO) promoted Primary Health Care; since the late 1990s and 2000s, the territorial expansion of residencies and the creation of Family Health Units (USF) have been promoted. In 2019, the Ministry of Public Health and Social Welfare (MSPBS) launched a strategic plan to train 1,200 specialists over 8 years and expand residency positions through CONAREM and new training units (25, 57–59).

Training is provided through a three-year residency with hospital rotations and an emphasis on primary and community care; regulation and accreditation are overseen by CONES/ANEAES, and since 2019 unified quality criteria have been established (58). There are 510 total positions (including residents), and in 2023, 664 family physicians certified by the SPMF were reported; this corresponds to 10.42 family physicians per 100,000 inhabitants in 2023 (25, 59). Family physicians work in public and private clinics, coordinate Family Health Units (USF), participate in emergency care, teaching and research, and hold managerial and administrative positions within the Primary Health Care strategy (25, 58).

### Peru

Family Medicine in Peru was formalized in the 1980s; in 1988 the Ministry of Health (MINSA), with international support, promoted the strengthening of Primary Health Care, and in 1989 the first three-year training programs were initiated, adapted to local needs (60). Since the 2000s, the specialty has gained momentum with the incorporation of family physicians into the MINSA and EsSalud networks, consolidating its role in primary care (60, 61).

Training is regulated by the National Committee for Medical Residency (CONAREME) and is provided through three-year residency programs; by 2023 there were more than 15 accredited programs and around 200 residents are trained per year (62). The curriculum combines clinical rotations (outpatient care, emergency care, pediatrics, and gynecology) with an emphasis on public health, epidemiology, and health management (62). According to the Peruvian Medical College, by 2025 there are 1,205 registered family physicians with a rate of 3.48 per 100,000 inhabitants, a figure considered insufficient and concentrated in urban areas; rural areas show a marked shortage, generating access gaps (60, 62, 63).

## Growth in family medicine workforce 2014–2024

To obtain an overview of workforce growth, information was compared over an interval of approximately 10 years. Because historical information was not uniformly available across countries, the baseline and follow-up years used for comparisons were not identical. Whenever possible, data points separated by approximately a decade were selected; however, the specific years varied according to the availability, completeness, and quality of national records. Therefore, the reported trends should be interpreted as indicative of medium-term workforce evolution rather than as strictly synchronized regional estimates.

Obtaining comparable rates of Family Medicine specialists per 100,000 inhabitants for Argentina, Colombia, Ecuador, Mexico, Paraguay, Panama, and Peru presents important methodological challenges due to the heterogeneity of sources, definitions, and reference years. Counts of specialists derive from diverse sources, including ministerial records, human resource observatories, professional colleges, scientific societies, academic publications, and institutional reports, which do not always apply the same inclusion criteria (e.g., certified specialists, registered specialists, active practitioners, or cumulative historical counts). Furthermore, the most recent available data differ across countries, requiring the use of population estimates corresponding to each reference year to calculate standardized rates and improve cross-country comparability (Table 3).

In this context, the figures presented for each country correspond to the most recent verifiable data identified from the most robust source available—preferably official sources, or alternatively professional and academic sources when updated public registries were unavailable. Specialist counts were paired with population estimates from the corresponding period to calculate rates per 100,000 inhabitants, while recognizing that differences in information systems, registry updating, and database coverage may generate variations compared with other published reports (Table 3).

Table 3, summarizes the historical development of Family Medicine across the seven countries, including the period of formal establishment of the specialty, changes in the number of specialists and specialist-to-population ratios, and trends in residency training capacity.

The comparative analysis demonstrates a generalized increase in the Family Medicine workforce throughout the region, although the magnitude and pace of growth varied substantially among countries. Ecuador and Paraguay experienced the largest relative increases in both the absolute number of specialists and specialist-to-population ratios, reflecting sustained efforts to expand residency training and strengthen PHC-oriented policies. Argentina also showed substantial growth, with its specialist rate more than doubling during the observed period. Mexico exhibited continued expansion from an already large workforce base, suggesting consolidation of an established specialty rather than an early growth phase. Colombia and Peru showed more gradual increases, consistent with progressive but slower workforce development processes.

Panama presented a comparatively low current specialist-to-population ratio and lacked a fully comparable historical baseline for workforce analysis. However, this finding should be interpreted within the context of the country’s relatively small population size and training capacity rather than as evidence of an incipient specialty. Family Medicine has been institutionally established in Panama for several decades and is supported by a nationally recognized residency training program and professional organizations.

Despite these advances, substantial differences remain among countries. Specialist availability ranges from fewer than 2 family physicians per 100,000 inhabitants in some settings to more than 17 per 100,000 inhabitants in others. Similarly, marked variation exists in residency training capacity, workforce production, and the degree of integration of Family Medicine into PHC delivery models. These findings suggest that, although Family Medicine has expanded throughout the region, its workforce development continues to reflect differences in health system organization, training policies, human resource planning, and the priority assigned to Primary Health Care within national health strategies (Table 3).

## Policy and practice implications

**Figure 1:**
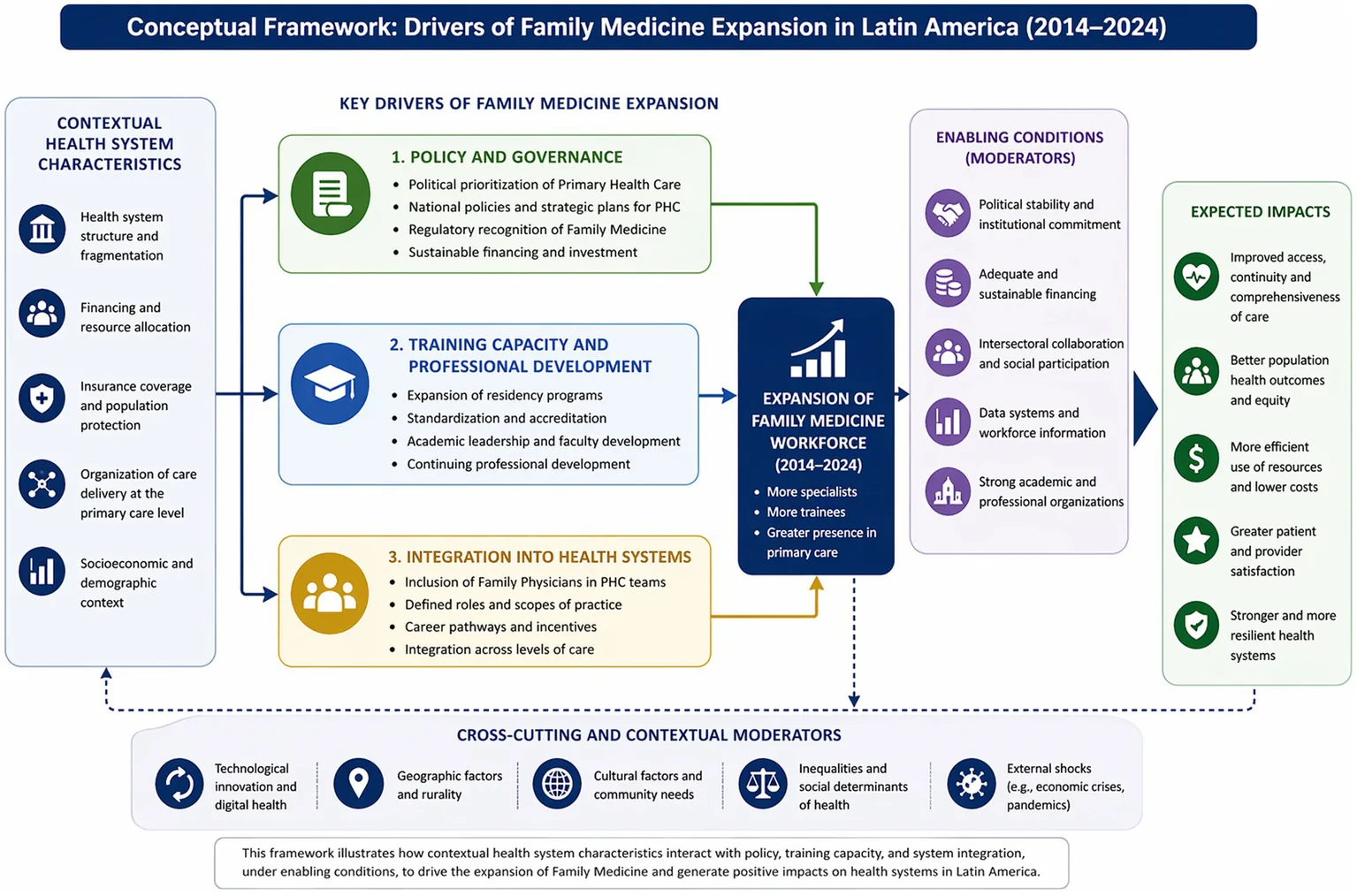
Conceptual framework: Drivers of family medicine expansion in Latin America (2014–2024). AI-generated image (OpenAI, ChatGPT, GPT-4), April 21, 2026 (75).

The results of this study suggest that strengthening Family Medicine in Latin America requires integrated strategies that go beyond increasing training positions (Figure 1).

Key policy priorities include:

- Expanding and decentralizing residency programs
- Strengthening incentives for equitable geographic distribution
- Integrating Family Medicine into PHC service delivery models as a true entry point
- Aligning workforce planning with health system organization and financing

The increase in specialists is associated with the expansion of residency positions, unified programs in some countries, and the active role of universities, ministries, and social security institutions. However, common obstacles persist, such as the segmentation and fragmentation of health systems, the geographic concentration of specialists, and shortcomings in the systematic documentation of the impact of family physicians on healthcare outcomes.

## Discussion

This study, developed by the IBIMEFA group, shows heterogeneous growth of the Family Medicine specialty in the seven countries studied (Argentina, Colombia, Ecuador, Mexico, Paraguay, Panama, and Peru) between 2014 and 2024. The increase in specialists is associated with the expansion of residency positions, unified programs in some countries, and the active role of universities, ministries, and social security institutions. However, common obstacles persist, such as the segmentation and fragmentation of health systems, the geographic concentration of specialists, and shortcomings in the systematic documentation of the impact of family physicians on healthcare outcomes.

These findings confirm and update previous work on the uneven progress of Family Medicine in Ibero-America (6, 25, 64). They are consistent with other reviews that point to the influence of international Primary Health Care frameworks (1960s–1970s) and with studies that highlight the central role of institutions such as IMSS, IESS, or pioneering universities in consolidating the specialty (39, 46, 65). Unlike some previous narrative series, this multinational study validated by experts allows for a more systematic comparison of policies and training supply among countries.

A cross-country comparison reveals several recurring patterns. First, the countries that achieved the greatest workforce expansion generally combined increases in residency positions with explicit policy commitments to Primary Health Care and active support from universities, ministries of health, and scientific societies (6, 25, 33, 42, 58). Second, sustained growth was facilitated by mechanisms linking specialist training to broader health system priorities, particularly in settings where Family Medicine was incorporated into PHC reform initiatives (3, 24, 25, 66). Third, despite differences in historical trajectories and health system structures, similar barriers were observed across countries, including fragmentation of service delivery, geographical maldistribution of specialists, insufficient workforce planning, and limited incorporation of family physicians as the preferred entry point to care (8, 9, 11, 14, 16, 25, 26, 36, 67). These findings suggest that the development of Family Medicine depends not only on training capacity but also on the degree of alignment between workforce policies and PHC-oriented health system reforms (25, 66, 68).

Although international evidence shows that systems with strong Primary Health Care achieve better health outcomes, lower premature mortality, and greater equity. Comparative experiences show consistent patterns: in Europe, territorial models with family physicians as the core achieve better control of chronic diseases and fewer avoidable hospitalizations (69); in Asia, universal coverage reforms supported by robust primary care networks have expanded access and cost containment (70); and in Africa, integrated community programs have improved maternal and child health indicators and basic access even in contexts with a shortage of physicians (71). In this context, strengthening Family Medicine is strategic, as these specialists contribute comprehensive clinical problem-solving capacity, continuity of care, and intersectoral coordination; the most cost-effective policies include expanding residency programs, labor incentives, multiprofessional teams, and standardized protocols for chronic diseases (66, 69).

International evidence indicates that systems with strong Primary Health Care achieve better outcomes and equity (69–72). Its effective implementation depends more on sustained and coherent political decisions than on isolated interventions; countries with lasting progress maintain institutional continuity, stable financing, and consistent regulatory frameworks (68). Successful reforms are cumulative processes associated with stable policies that allow the consolidation of networks, human resource training, and public trust (68, 73). When policies are discontinuous, systems tend to fragment and lose problem-solving capacity even with financial investment.

These findings highlight the need for integrated policy approaches to strengthen Family Medicine within Primary Health Care systems in Latin America. Expanding residency training positions is essential to address current workforce gaps; however, this effort must be accompanied by stronger governance of PHC to ensure coherent implementation of policies, sustained financing, and effective coordination across health system levels. In addition, aligning workforce planning with service delivery needs is critical to ensure that the training and deployment of Family Medicine specialists respond to population health demands and contribute to improving access, continuity, and quality of care.

One of the most relevant findings of this study is the distinction between the expansion of Family Medicine as a medical specialty and its institutional integration within health systems. Although all countries analyzed showed growth in the number of family physicians, this expansion was not consistently accompanied by population assignment mechanisms, strong gatekeeping functions, or the consolidation of Family Medicine as the main entry point to care. These findings suggest that increasing the number of specialists, while necessary, may be insufficient to strengthen Primary Health Care if broader organizational, regulatory, and financing reforms are not implemented. Workforce development and health system transformation appear to be related but distinct processes, requiring coordinated policy action to ensure that the competencies of family physicians can effectively contribute to continuity, comprehensiveness, and coordination of care.

Beyond workforce expansion and financing mechanisms, the long-term sustainability of Family Medicine also depends on professional and cultural factors. Recent qualitative research in Ibero-America has shown that the professional identity of family physicians is built during residency training and strengthened through clinical practice, teaching activities, and sustained engagement with families and communities. Family Medicine specialists identify continuity of care, comprehensiveness, community commitment, and person-centered care as defining elements of their professional role. These findings suggest that strengthening Family Medicine requires not only increasing training capacity but also promoting educational environments and service models that reinforce the distinctive identity and social value of the specialty (74).

This study presents some limitations that should be considered when interpreting the results. First, the selection of countries and sources was not probabilistic, and data availability varied across contexts, which limits the temporal comparability of the figures. Second, heterogeneity in professional titles and the variable quality of national records required, in some cases, the use of gray literature and key informants, which may introduce reporting biases. Finally, the descriptive and cross-sectional design prevents establishing causal relationships between policies and the increase in specialists and limits the evaluation of the real impact of family physicians on health performance indicators.

Among the main strengths, it stands out that this is a multinational and collaborative study with validation by IBIMEFA experts, which integrated and triangulated official sources, academic literature, and gray evidence to improve the relevance and contextualization of the data. In addition, the comparative approach allowed the identification of regional patterns and national particularities relevant for policy formulation and the planning of training in Family Medicine.

## Conclusion

In the seven countries studied, the presence of specialists in Family Medicine increased, with the most significant increases occurring in Ecuador and Paraguay; Mexico and Argentina showed greater stability due to their larger existing base, while Colombia, Peru, and Panama progressed more slowly, possibly due to less clear or effective policy decisions.

For Family Medicine to fully contribute to strengthening Primary Care, in addition to expanding training opportunities, integrated policies are required that coordinate training, financing, employability, and service organization. At the same time, the sustainability of the specialty requires strengthening professional identity, community engagement, and the recognition of Family Medicine as a central component of health systems Furthermore, it is necessary to systematically document its impact on quality, problem-solving capacity, and equity through evaluative studies that guide the implementation of these policies.
